# Life events as predictors of unsustainable working life trajectories from a life course perspective

**DOI:** 10.1093/eurpub/ckac129.093

**Published:** 2022-10-25

**Authors:** M Wang, A Raza, J Narusyte, K Silventoinen, P Böckerman, P Svedberg, A Ropponen

**Affiliations:** 1 Department of Clinical Neuroscience, Karolinska Institutet, Stockholm, Sweden; 2 Center of Epidemiology and Community Medicine, Stockholm County Council, Stockholm, Sweden; 3 Population Research Unit, Faculty of Social Sciences, University of Helsinki, Helsinki, Finland; 4 School of Business and Economics, University of Jyväskylä, Jyväskylä, Finland; 5 Labour Institute for Economic Research, Helsinki, Finland; 6 IZA Institute of Labor Economics, Bonn, Germany; 7 Finnish Institute of Occupational Health, Helsinki, Finland

## Abstract

**Background:**

The association between family-related life events (e.g., getting married or having children) and unsustainable working life in terms of unemployment, sickness absence and disability pension (SA/DP) are rarely studied from a life-course perspective although having public health importance. We investigated trajectories of unsustainable working life, and the associations between change in family-related life events and unsustainable working life trajectories by controlling for familial factors.

**Methods:**

This is a prospective cohort study of 37,867 Swedish twins aged between 20-40 years on 31st December 1994. Data on trajectories of annual unemployment, SA/DP, and a combined measure of unsustainable working life months was collected from the Swedish national registers. The trajectories over a 23-year period were analysed by group-based trajectory modelling. Associations of change in family-related life events with trajectory groups in the whole sample were estimated by multinomial logistic regression and in discordant twin pairs (n = 4,647 pairs) with conditional models.

**Results:**

Most participants had no or low levels of unemployment, SA/DP or combined unsustainable working life during 1994-2016. Individuals who were stably married or changed from being single living without children to married living with children had a decreased risk of unsustainable working life compared to individuals with stable family-related life events. The risk of unsustainable working life months over time was higher among individuals who changed from married to single status regardless of having children (range of HRs:1.31-4.44).

**Conclusions:**

Family-related life events such as maintaining the relationship or getting married and having children decreases the risk of unsustainable working life while divorce is a risk factor for unsustainable working life. From a public health perspective, actions to support family formation or life would consequently promote a sustainable working life.

**Key messages:**

• Unsustainable working life was less likely among married and among those who changed from single living without children to married with children compared to those with stable family life events.

• Individuals who changed from being married to divorced status had an increased risk of unsustainable working life over time and therefore being potentially an important group for public health.

